# Prenatal delta-9-tetrahydrocannabinol exposure is associated with changes in rhesus macaque DNA methylation enriched for autism genes

**DOI:** 10.1186/s13148-023-01519-4

**Published:** 2023-07-06

**Authors:** Lyndsey E. Shorey-Kendrick, Victoria H. J. Roberts, Rahul J. D’Mello, Elinor L. Sullivan, Susan K. Murphy, Owen J. T. Mccarty, Danny J. Schust, Jason C. Hedges, A. J. Mitchell, Jose Juanito D. Terrobias, Charles A. Easley, Eliot R. Spindel, Jamie O. Lo

**Affiliations:** 1 Division of Neuroscience, Oregon National Primate Research Center, Oregon Health and Science University, Beaverton, OR 97006 USA; 2 Division of Reproductive and Developmental Sciences, Oregon National Primate Research Center, Oregon Health and Science University, Beaverton, OR 97006 USA; 3 Department of Obstetrics and Gynecology, Division of Maternal Fetal Medicine, Oregon Health and Science University, Portland, OR 97239 USA; 4 Department of Psychiatry, Oregon Health and Science University, Portland, OR 97239 USA; 5 Department of Behavioral Neuroscience, Oregon Health and Science University, Portland, OR 97239 USA; 6 Department of Obstetrics and Gynecology, Duke University Medical Center, Durham, NC 27701 USA; 7 Department of Biomedical Engineering, Oregon Health and Science University, Portland, OR 97239 USA; 8 Department of Urology, Oregon Health and Science University, Portland, OR 97239 USA; 9 Department of Environmental Health Science, University of Georgia College of Public Health, Athens, GA 30602 USA

**Keywords:** Delta-9-tetrahydrocannabinol (THC), Cannabis, Marijuana, DNA methylation, Prenatal substance use

## Abstract

**Background:**

With the growing availability of cannabis and the popularization of additional routes of cannabis use beyond smoking, including edibles, the prevalence of cannabis use in pregnancy is rapidly increasing. However, the potential effects of prenatal cannabis use on fetal developmental programming remain unknown.

**Results:**

We designed this study to determine whether the use of edible cannabis during pregnancy is deleterious to the fetal and placental epigenome. Pregnant rhesus macaques consumed a daily edible containing either delta-9-tetrahydrocannabinol (THC) (2.5 mg/7 kg/day) or placebo. DNA methylation was measured in 5 tissues collected at cesarean delivery (placenta, lung, cerebellum, prefrontal cortex, and right ventricle of the heart) using the Illumina MethylationEPIC platform and filtering for probes previously validated in rhesus macaque. In utero exposure to THC was associated with differential methylation at 581 CpGs, with 573 (98%) identified in placenta. Loci differentially methylated with THC were enriched for candidate autism spectrum disorder (ASD) genes from the Simons Foundation Autism Research Initiative (SFARI) database in all tissues. The placenta demonstrated greatest SFARI gene enrichment, including genes differentially methylated in placentas from a prospective ASD study.

**Conclusions:**

Overall, our findings reveal that prenatal THC exposure alters placental and fetal DNA methylation at genes involved in neurobehavioral development that may influence longer-term offspring outcomes. The data from this study add to the limited existing literature to help guide patient counseling and public health polices focused on prenatal cannabis use in the future.

**Supplementary Information:**

The online version contains supplementary material available at 10.1186/s13148-023-01519-4.

## Background

Epigenetic regulation and modulation of gene expression are essential for normal fetal development [1]. Chromatin remodeling through DNA methylation is one of the most studied epigenetic processes. The epigenome, and particularly DNA methylation, is responsive to exogenous exposures and can elicit gene dysregulation, which may influence cellular function and tissue development [2]. DNA methylation is a critical mediator of normal placental [3] and fetal development, including the central nervous system [4, 5]. Epigenetic responses to maternal psychoactive substance use may lead to long-term molecular alterations implicated in addiction and psychiatric disorders [6, 7]. Cannabis, commonly known as marijuana, refers to a group of plants with psychoactive and medicinal properties. Use of cannabis and cannabis-derived products in the USA has increased as a greater number of states have legalized both recreational and medicinal cannabis use, which has resulted in diminished public perception of potential physiological risks of cannabis use [8]. Cannabis use among pregnant individuals [9], especially during the critical developmental window in the first trimester to mitigate morning sickness symptoms, has doubled in the past decade [10] and half of those that use cannabis will continue to use throughout pregnancy [11–13].

Cannabis acts by targeting the endocannabinoid system to exert neuromodulatory and paracrine effects, in part via epigenetic modifications, that impact organogenesis, neurogenesis, and gliogenesis [7, 14]. The main psychoactive cannabinoid delta-9-tetrahydrocannabinol (THC) mimics endogenous cannabinoids by crossing the placenta and binding to cannabinoid receptors, CB1 and CB2, in the placenta and major fetal organs, including the brain [15–20], leading to concern for detrimental fetal and offspring outcomes [21–24].

Although limited in number and mechanistic understanding, studies suggest adverse effects of prenatal cannabis exposure that include preterm birth, stillbirth and small for gestational age infants [24–28]. Maternal cannabis use has also been associated with an increased risk for neurobehavioral morbidity in human offspring, including autism spectrum disorder (ASD), attention deficit hyperactivity disorder (ADHD), intellectual disability and learning disorders, and other neuropsychiatric disorders [29–35]. Preliminary data in humans, mice and rats indicate that prenatal cannabis exposure results in altered DNA methylation in both the placenta and the brains of exposed offspring and that these epigenetic marks may be a potential mechanistic link between maternal cannabis use and associated negative offspring outcomes [36–38]. Prenatal cannabinoid exposure has also resulted in increased anxiety-like behavior and altered genome-wide brain DNA methylation in mouse offspring [38].

Although there are limited clinical data regarding the effects of cannabis exposure during pregnancy, the rising prevalence of prenatal cannabis use [39] is of significant concern, as the potential short-term medicinal benefits for nausea and pain may be outweighed by the possibility of longer-term adverse impacts to offspring. Despite its potential adverse impact, there is a striking paucity of in vivo data on the effects of THC on fetal development in part because of the regulatory challenges, heterogeneity, and confounds in human studies. In addition, despite evidence suggesting that maternal substance use can impact the fetal epigenome [14, 37], our understanding of the impact of prenatal cannabis exposure on placental and fetal epigenetic regulation is impeded by the limitations and feasibility of tissue sampling.

To address key gaps in the evidence and overcome the limitations of previous human studies, we developed a novel non-human primate (NHP) model of chronic cannabis exposure via edible THC consumption [40, 41]. The NHP is a strong translational model that recapitulates human gene expression and regulation during development and has a similar fetal ontogeny [42, 43], placental structure [42, 43], and THC plasma disposition to humans. Use of a NHP model overcomes the limitations of previous human studies by facilitating tissue sampling [44], resulting in observations that are directly translatable to human pregnancies [45]. Edibles are the second most common mode of cannabis delivery [46] and are often recommended by dispensaries to pregnant individuals for nausea [47]. Our NHP model of THC edible consumption uniquely recapitulates typical human use, minimizes subject variability and allows precise THC dosing to elucidate direct biological consequences of chronic prenatal cannabis exposure, while methodically controlling for potential confounders.

The incidence of cannabis use is rising, and there is a critical need for investigation of the effects of in utero exposure to cannabis at the molecular level, in order to increase our understanding of the risks on fetal development, the potential underlying mechanisms, and future health outcomes. Our objective was to use our novel NHP model of maternal THC edible use to help bridge this knowledge gap by determining the impact of prenatal THC exposure on the placental and offspring epigenome.

## Results

### Study sample characteristics

Tissues were collected following cesarean section delivery during the third trimester at gestational day 155 (term is ~ 168 days) following chronic prenatal THC-exposure (THC, *n* = 5) from preconception throughout gestation or placebo-exposure (CON, *n* = 5). There were no significant differences in baseline characteristics between THC- and CON-exposed dams, placental, or offspring birth characteristics as shown in Table 1. DNA methylation was measured using the Infinium Methylation EPIC platform in all available tissues. Heart DNA was not available for one THC-exposed animal, and one cerebellum dataset (CON) was removed following quality control analysis due to low intensity. After quantile normalization and retaining probes previously validated in *Macaca mulatta* [48], we observed clear separation of datasets by tissue (Additional file 1: Figure S1).

**Table 1:** Demographics and sample characteristics by treatment group

Characteristic	Control	THC	*p* value
n	5	5	
Maternal age (years)	10.0 ± 2.3	9.9 ± 2.1	0.945
Parity	3.6 ± 0.5	2.2 ± 2.2	0.226
Maternal weight at G60 (kg)	7.53 ± 1.02	7.74 ± 0.47	0.692
Maternal weight at delivery (G155) (kg)	8.94 ± 1.20	8.95 ± 0.48	0.990
Fetal birth weight (kg)	0.48 ± 0.08	0.46 ± 0.07	0.676
Total placental weight (g)	105.2 ± 17.6	105.0 ± 18.7	0.984
Fetal sex (male:female)	2:3	3:2	–-

Data are means ± SD. Statistical analysis performed using Welch’s *t* test

### Prenatal delta-9-tetrahydrocannabinol exposure is associated with changes in placental and offspring tissue DNA methylation

**Fig. 1:**
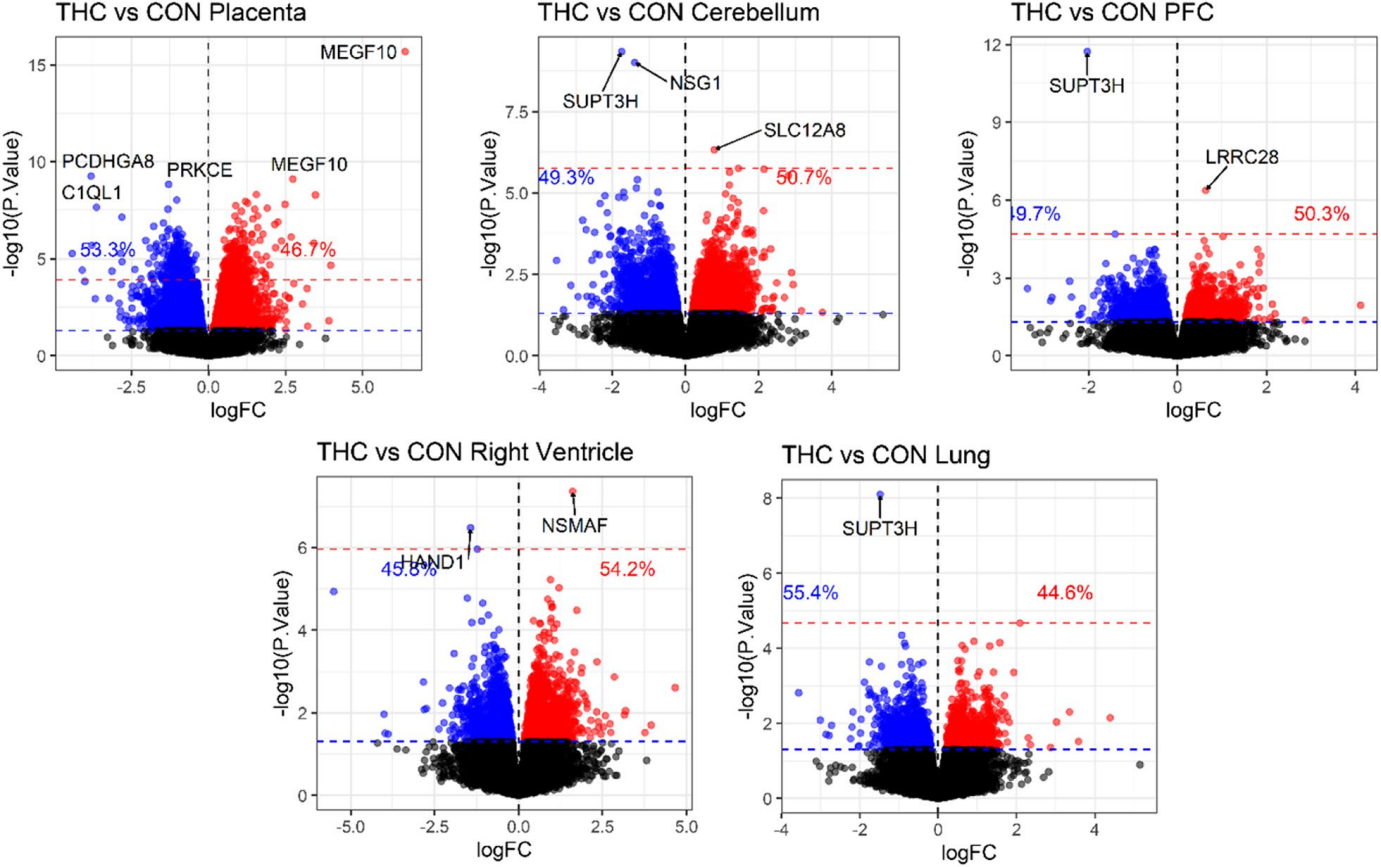
Prenatal THC is associated with differential methylation at individual CpGs in each tissue tested. Volcano plots for each tissue show CpG-specific associations with THC exposure using limma with a factorial design of group and tissue, accounting for within-subject correlation. Each plot depicts the logFC for THC versus CON on the x-axis, − log10(*p* values) on the y-axis. Percentages indicate relative proportions of nominally significant hypomethylated (blue) and hypermethylated (red) CpGs. The dotted blue line indicates nominal *p* < 0.05, and the dotted red line indicates CpGs reaching significance after adjusting for multiple comparisons using the Benjamini and Hochberg method to control false discovery rate (FDR)

**Fig. 2:**
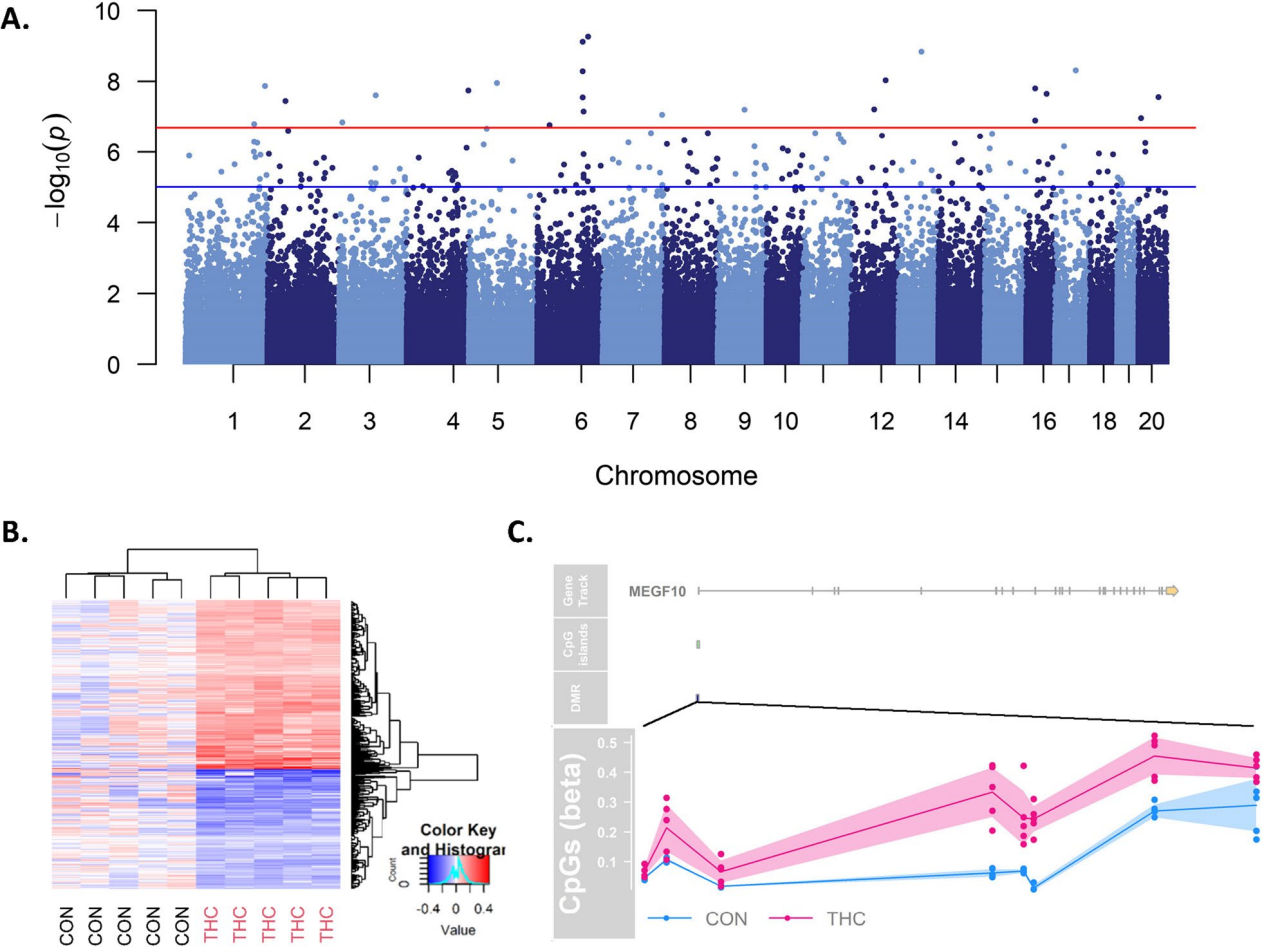
Placental methylation is significantly associated with THC exposure across the genome. **A** Manhattan plot of CpGs in placenta associated with prenatal THC exposure with the uncorrected log10 (*p* values) plotted on the y-axis and chromosomal location on the x-axis (Mmul_10). The blue line is drawn to indicate CpGs that surpassed *p* value < 1e−5, a threshold for suggestive association, and the red line is to highlight CpGs that passed a stringent Bonferroni threshold of *p* value < 2e−07. **B** Heatmap of placental DMCs passing FDR significance (*n* = 573 CpGs) clearly separate by treatment group. Values represent the log-transformed and mean centered average methylation values per CpG. **C** Prenatal THC is associated with methylation changes in placental *MEGF10* promoter. Gviz plot [4] showing from top to bottom the gene structure, CpG island proximity, and individual CpG beta values for each animal (THC animals are shown in pink and Control in blue)

We tested for differentially methylated CpGs (DMC)s with prenatal THC exposure in each tissue using 234,836 CpG-specific linear models with factorial design of group and tissue, accounting for within-subject correlation. Genomic inflation factors from the tissue-specific contrasts ranged from lambda = 0.47–1.01 (Additional file 1: Figure S2). A total of 581 CpGs were significantly associated with prenatal THC exposure after adjusting for multiple comparisons using the Benjamini and Hochberg method to control false discovery rate (FDR) (Fig. 1) [49]. The top 5 DMCs per tissue are presented in Table 2, and the full list of FDR significant (< 0.05) DMCs is available in Additional file 2: Table S1. Out of 581 FDR DMCs, 573 (98%; 335 hypermethylated/ 238 hypomethylated) were identified in placenta and appeared to be dispersed across the genome with the exception of a spike in association located at chromosome 6 based on rhesus annotation (Mmul_10; Fig. 2A). Hierarchical clustering of placental DMCs (Fig. 2B) and a MDS plot of the most variable CpGs (Additional file 1: Figure S3) showed clear separation of THC versus CON animal DNA methylation in the placenta. The top CpG associated with THC exposure, cg23018092, was specific to placental tissue (adjusted *p* value = 4.85e−11; logFC = 6.38), located at chr6:124772737 in the rhesus genome. Of note, cg23018092 mapped to a CpG island overlapping the first exon of *MEGF10* and there were 5 additional CpGs within this CpG island all hypermethylated with THC exposure at FDR significance (Fig. 2C).

**Table 2:** Top five DNA methylation loci per tissue associated with prenatal THC exposure

	Probe	Mmul_10		Nearest gene	logFC	*p* value	Adj. *p* val
		Chr	Pos				
**Placenta**	**cg23018092**	6	124,772,737	***MEGF10***	6.38	**2.06E−16**	**4.85E−11**
	**cg27346707**	6	124,772,721	***MEGF10***	2.74	**7.65E−10**	**5.99E−05**
	**cg05494467**	6	138,890,510	***PCDHGA8***	**− **3.81	**5.40E−10**	**5.99E−05**
	**cg20825437**	13	63,151,971	***PRKCE***	**− **1.30	**1.45E−09**	**8.54E−05**
	**cg03721362**	6	124,772,671	***MEGF10***	3.47	**5.12E−09**	**0.0002**
**Cerebellum**	**cg00617180**	4	124,660,978	***SUPT3H***	**− **1.75	**4.49E−10**	**0.000105**
	**cg19948549**	5	8,503,869	***NSG1***	**− **1.39	**9.65E−10**	**0.000113**
	**cg17478992**	2	151,164,369	***SLC12A8***	0.78	**4.69E−07**	**0.036697**
	cg13699963	16	69,873,069	*SDK2*	2.14	**1.86E−06**	0.08718
	cg11031701	16	35,959,387	*TBX2*	1.44	**1.74E−06**	0.08718
**Prefrontal Cortex**	**cg00617180**	4	124,660,978	***SUPT3H***	**− **2.04	**1.87E−12**	**4.38E−07**
	**cg01898225**	7	78,635,212	***LRRC28***	0.63	**4.18E−07**	**0.049039**
	cg02616023	1	88,639,226	*NOS1AP*	**− **1.40	**2.05E−05**	0.999987
	cg14964115	9	115,397,494	*FAM160B1*	1.02	**2.48E−05**	0.999987
	cg13915892	13	1,072,439	*LMAN2L*	0.60	**3.62E−05**	0.999987
**Heart**	**cg22754569**	8	59,305,442	***NSMAF***	1.61	**4.36E−08**	**0.010244**
	**cg19504702**	6	151,889,356	***HAND1***	**− **1.43	**3.34E−07**	**0.039244**
	cg00617180	4	124,660,978	*SUPT3H*	**− **1.23	**1.10E−06**	0.085922
	cg03396151	7	13,532,030	*MEIS2*	0.95	**6.03E−06**	0.353767
	cg06933370	7	13,532,249	*MEIS2*	1.21	**9.42E−06**	0.442549
**Lung**	**cg00617180**	4	124,660,978	***SUPT3H***	**− **1.48	**7.89E−09**	**0.001852**
	cg17760405	4	72,836,058	*SIM1*	2.08	**2.11E−05**	0.999998
	cg10762132	13	15,816,921	*SLC20A1*	**− **0.94	**4.46E−05**	0.999998
	cg23616524	3	156,111,969	*UBE2H*	0.91	**6.44E−05**	0.999998
	cg11126485	14	12,810,784	*TMEM138*	1.57	**7.09E−05**	0.999998

Tested using limma with CpG-wise statistical models with factorial design of group and tissue, accounting for within subject correlation

Bold text indicates FDR adjusted *p*-value < 0.05

In fetal brain, we assessed DNA methylation of the cerebellum and prefrontal cortex (PFC), separately. Three CpGs reached FDR significant (< 0.05) association with THC exposure in cerebellum annotated to *SUPT3H, NSG1*, and SLC12A8; and 2 in PFC annotated to *SUPT3H* and *LRRC28* (Fig. 1; Table 2). In the right heart ventricle, we identified 2 FDR significant DMCs annotated to *NSMAF* and *HAND1* (Fig. 1; Table 2). In fetal lung, 1 CpG reached FDR significance annotated to *SUPT3H.* However, this CpG (cg00617180) was 1 bp upstream of a common single nucleotide polymorphism (SNP) in our rhesus macaque colony (allele frequency = 0.567) and showed a consistently large effect size across all tissues, suggesting a potentially genetic, rather than treatment effect, at this loci (Additional file 1: Figure S4A).

Given the larger number of CpGs differentially methylated with THC exposure in placental DNA, we also extracted and annotated placental differentially methylated regions (DMRs). We identified 403 significant placental THC-DMRs after FDR correction (Additional file 2: Table S2). Consistent with individual CpG analysis results, the top DMR was annotated to *MEGF10* on chr5 (based on EPIC hg19 annotation; chr6 in the rhesus genome) and included 8 CpGs with a mean increase in methylation of 23% with THC exposure. The second and third most significant DMRs ranked by Fisher’s multiple comparison statistic were located on the same chromosome and mapped to *ADAMTS19* and a large cluster of protocadherin genes, respectively (Additional file 1: Figure S5A; Additional file 2: Table S2).

### Gene ontologies (GO) and biological pathways in genes differentially methylated with THC

**Fig. 3:**
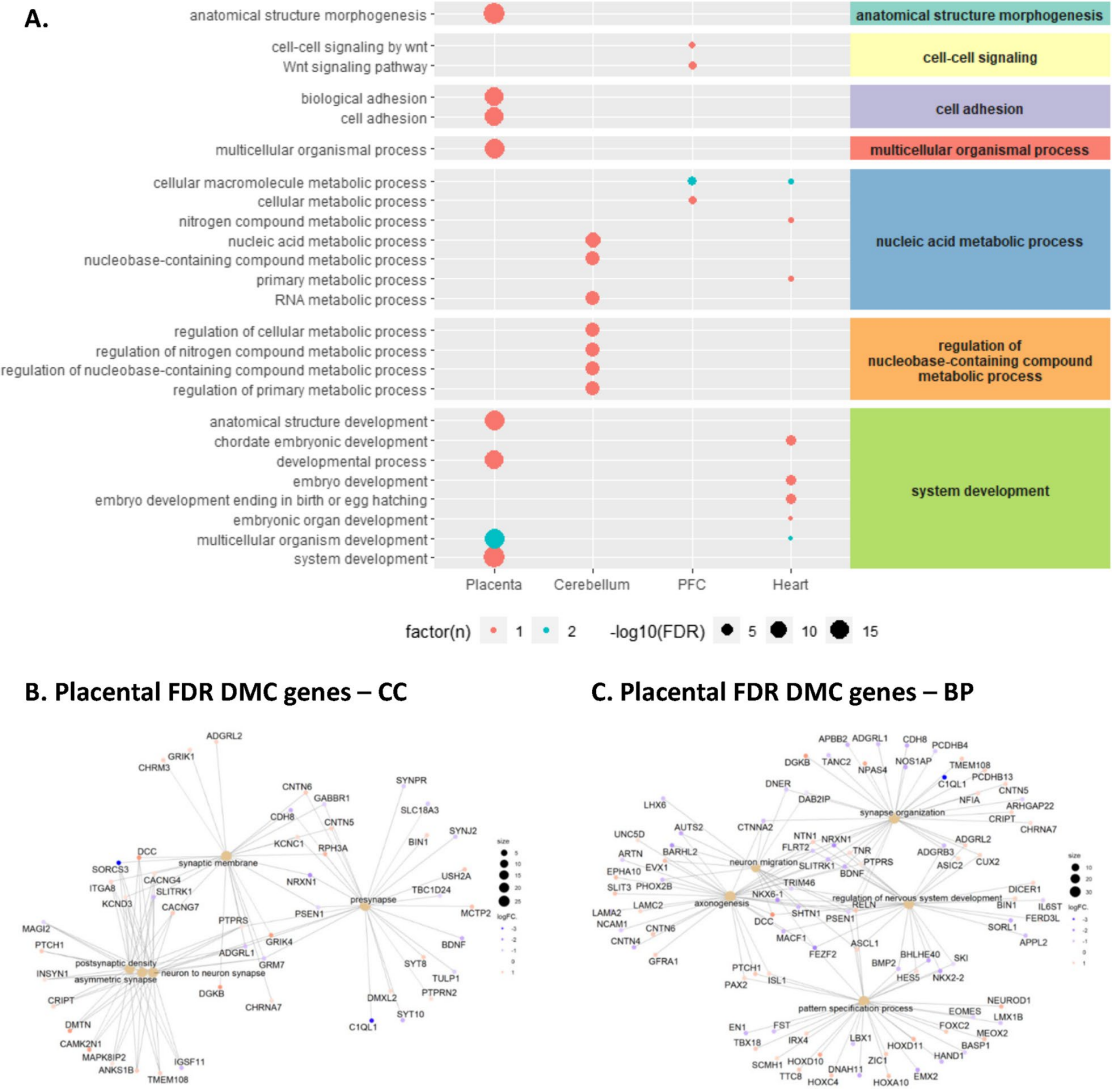
DMC genes are enriched for morphogenesis, nucleic acid metabolism, and nervous system development related terms. **A** The top 10 FDR significant GO terms in the biological processes category are presented for each tissue clustered according to larger parent categories identified using rrvgo. Color indicates the number of tissues enriched for each term and the size of circles indicates − log10 FDR *p* value across terms. There was no FDR significant enrichment of GO terms in lung tissue. In the placenta, the clusterProfiler R package was used to visualize enriched cellular compartments (**B**), biological processes (**C**), and connected genes using the more stringent set of 573 FDR significant DMCs

We next identified enriched Gene ontology (GO) terms and Kyoto encyclopedia of genes and genomes (KEGG) pathways within each tissue using the missMethyl package which adjusts for bias related to gene length and probe density [50]. Given our sample size, we included DMCs with a nominal *p *value < 0.05 in order to inform future functional studies. Top FDR significant GO biological processes per tissue are shown in Fig. 3A, and the full list of FDR significant terms and pathways is presented in Additional file 2: Table S3. In placenta, there were 549 significant GO terms after multiple testing correction, and the top enriched biological processes among placental DMCs included several terms related to development and morphogenesis including “anatomical structure morphogenesis” and “system development”, while “calcium signaling pathway” was the top KEGG term. In the brain, we observed enrichment of terms related to “nucleic acid metabolic process” and “cell–cell signaling by wnt”, while heart tissue was enriched for terms related to system development (e.g., “embryo development” and “tube development”) and also “nucleic acid metabolic process”. There were no FDR significant GO terms in lung tissue. In the placenta only, we also tested for enrichment using the more stringent set of 573 FDR significant DMCs and the clusterProfiler R package to visualize enriched cellular compartments, biological processes, and annotated genes. Placental FDR DMCs were enriched for cellular compartments and biological process terms related to neuronal synapse, nervous system development, neuron migration, and axonogenesis (Fig. 3B, C).

### Prenatal THC changes in DNA methylation are enriched for candidate autism genes

**Fig. 4:**
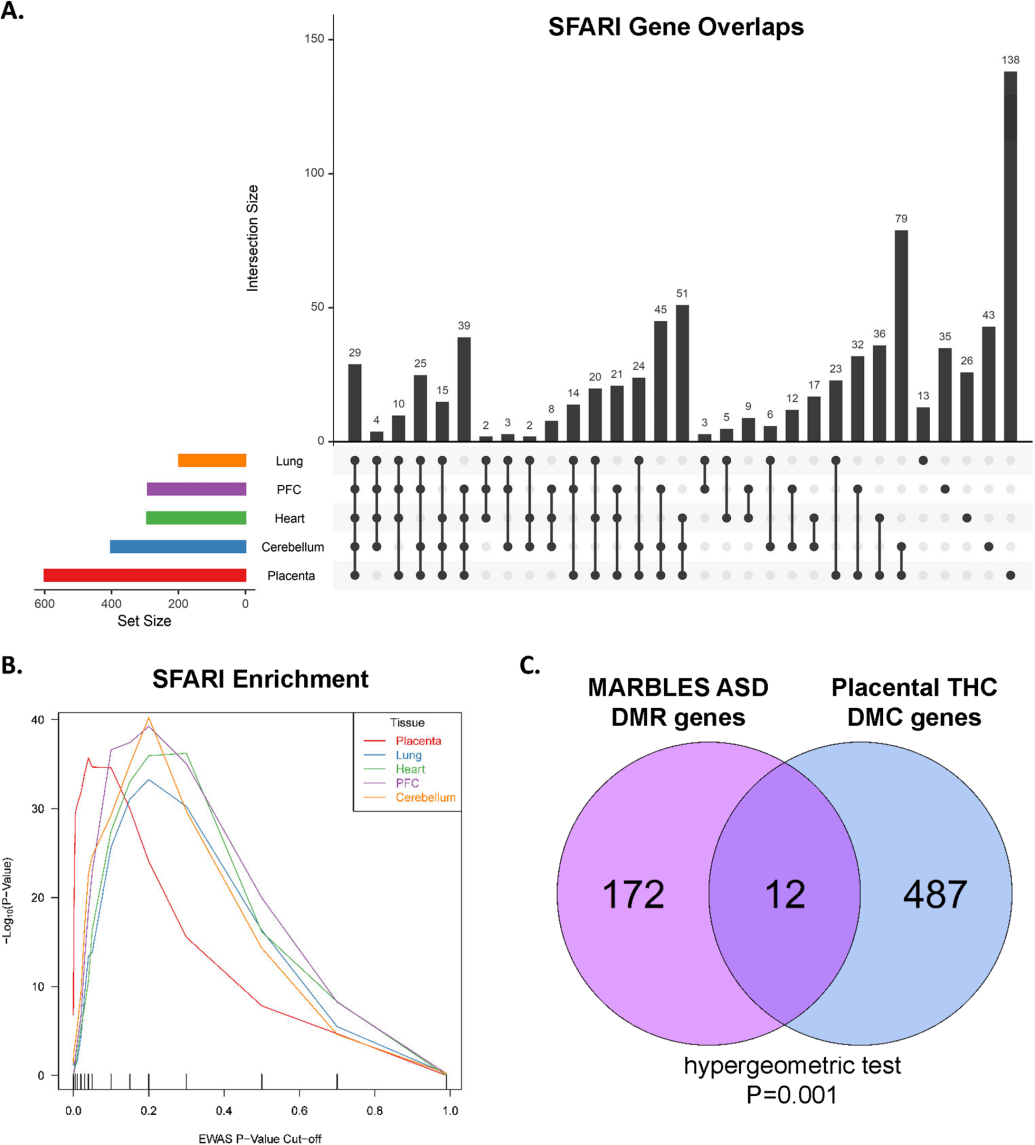
Candidate autism spectrum disorder genes are enriched among prenatal THC-DMCs for each tissue type. **A** UpSet plot of the overlap of SFARI genes annotated to one or more DMC for each tissue. The y-axis represents the number of intersections for each combination of sets across the x-axis. Twenty-nine SFARI genes were represented in DMC lists across all five tissues. **B** THC-DMC-associated genes were tested for enrichment for SFARI genes across a range of *p* value thresholds. The x-axis indicates *p* value threshold for association and the y-axis indicates the − log10 *p* value for enrichment at each threshold. Line colors represent unique tissues. **C** Venn diagram demonstrates the overlap of genes annotated to ASD DMRs in a prospective birth cohort study (MARBLES) with placental THC DMC genes. The hypergeometric test was used to test for significance of overlap

Based on prior reports suggesting an association between prenatal cannabis exposure and offspring risk of ASD [30, 51], we next tested for enrichment of candidate ASD genes among THC-exposure DMCs. From the Simons Foundation Autism Research Initiative (SFARI) database [52], there were 925 candidate ASD genes which annotated to one or more CpG included in our differential methylation analysis. We tested for enrichment at multiple p-value thresholds and performed permutation testing of random gene sets as a negative control. Twenty-nine candidate ASD genes were differentially methylated with THC in all tissues at nominal *p* < 0.05 (Fig. 4A) and 789 candidate ASD genes (85%) were annotated to 1 or more DMC in any tissue (Additional file 2: Table S4). In each of the 5 tissues analyzed, there was significant enrichment of SFARI genes annotated to DMCs at multiple *p*-value thresholds, with the greatest enrichment in placenta (Fig. 4B; Table 3). Permutation testing with random gene sets the same size as the SFARI list were not enriched among DMC genes (Additional file 1: Figure S6). Additionally, we compared our placental THC-DMC gene list to a list of differentially methylated regions (DMRs) in human placentas from pregnancies where the newborn was later diagnosed with ASD [53]. We found significant enrichment of human ASD-associated DMR genes among our FDR significant placental THC DMCs (Fig. 4C), which included 12 genes overlapping between the two datasets.

**Table 3:** SFARI gene enrichment at multiple *p* value thresholds

Threshold	Measure	Placenta	Lung	PFC	Heart	Cerebellum
*p* < 1e−04	Actual	49	2	0	1	5
	Expected	21.9	0.4	0.5	1.0	1.9
	OR	2.5	5.7	0.0	1.0	2.9
*p* < 0.001	Actual	143	5	2	3	19
	Expected	69.1	1.8	2.6	4.4	9.2
	OR	2.4	3.0	0.7	0.7	2.2
*p* < 0.005	Actual	285	13	23	28	56
	Expected	149.3	8.3	13.2	20.3	34.0
	OR	2.4	1.6	1.8	1.4	1.8
*p* < 0.01	Actual	356	27	49	57	102
	Expected	203.9	17.2	28.7	38.9	65.1
	OR	2.3	1.6	1.8	1.5	1.7
*p* < 0.05	Actual	601	198	291	294	402
	Expected	417.4	116.5	167.9	188.0	257.4
	OR	2.3	2.0	2.2	1.9	2.1

Statistical analysis performed using a chi-squared test statistic [54]

*PFC* prefrontal cortex

### Differential methylation signatures following prenatal THC are associated with RNA expression

We next investigated the potential regulation of placental mRNA expression by DNA methylation using expression quantitative trait methylation (eQTM) analysis. We performed crude association between levels of 631 mRNA transcripts differentially expressed (DE) following prenatal THC with methylation levels at flanking CpGs within ± 250 kb. We tested correlation between 52,474 CpG-transcript pairs, of which 3309 individual CpGs were significantly correlated to 520 unique DE genes. The top DE gene was *DNAJA4* and was negatively correlated with methylation of 12 CpGs including the top eQTM CpG cg21248347 (*p* value = 7.05–06; Beta = − 20.67). Of note, the top differentially methylated gene is this study, *MEGF10*, did not meet differential expression criteria for inclusion in eQTM analysis, although we observed a trend for lower expression in the THC group relative to CON (log2FC = − 1.08; *p* value = 0.21). Interestingly, differential expression of several placental transcripts was associated with large numbers of unique CpGs. For example, 329 unique CpGs were significantly associated with the expression of 6 genes in a 2 megabase (Mb) block of chromosome 4 (equivalent to the 6p21.33 chromosome region of the human genome). This region included 99 CpGs associated with *CFB*, 61 with *GPANK1,* 56 with *NOTCH4,* 48 with *SKIV2L,* 46 with *PPP1R18,* and 19 with *VWA7* (Additional file 2: Table S5). Protein–protein interaction (PPI) networks of significant placental eQTM genes identified 73 enriched GO biological processes, including terms related to tissue development and response to external stimuli (Additional file 2: Table S6). Visual inspection of network connectivity suggested *SMAD3, JUN* and *HSP90* proteins as candidate network hubs with high confidence (Additional file 1: Figure S7). Additionally, we observed increased expression of *SHANK3*, which correlated with DNA methylation at several CpGs upstream of a large hypomethylated block identified in a prospective ASD cohort DMR analysis [53] (Additional file 1: Figure S8).

## Discussion

Our multi-tissue study demonstrates the utility of our rhesus macaque model to study the impact of prenatal THC use on fetal development and future offspring health, and to investigate the underlying molecular mechanisms. We focus our discussion on several key findings. First, differential methylation following THC exposure was present in all 5 tissue types, with the greatest effect measured in placenta. Second, prenatal THC exposure altered placental and fetal tissue epigenetic signatures at genes involved in embryonic development that may influence longer-term offspring outcomes. Third, changes in methylation in the placenta were associated with changes in RNA expression in genes enriched for tissue development and morphogenesis-related processes. Fourth, loci differentially methylated with THC were enriched for candidate ASD genes in all tissues. Lastly, we found significant overlap at the gene level when comparing differentially methylated loci in THC-exposed placentas from this study with DMRs in human placentas from pregnancies where the newborn was later diagnosed with ASD.

A total of 573 FDR (< 0.05) DMCs and 403 DMRs were associated with THC exposure in placenta. The top DMC and DMR in placenta were annotated to *MEGF10* (multiple EGF like domains 10), a gene highly expressed in brain tissue that encodes a protein critical to synaptic number and function in postnatal brain [55]. Interestingly, SNPs in the transcription regulatory region of *MEGF10* have been associated with increased risk of autism in a Chinese Han cohort, and *MEGF10* expression was lower in peripheral blood of autistic individuals compared to healthy controls [55]. In this study, we observed increased methylation over the transcription start site with THC exposure in placental tissue, which would suggest potential downregulation of expression. However, *MEGF10* expression in our placental RNA-seq dataset was relatively low in all placental samples, in agreement with the Human Protein Atlas [56]. Therefore, although we observed lower expression in the THC group relative to CON, the difference was not statistically significant (log2FC = − 1.08; *p* value = 0.21). Further work is necessary to determine the functional significance of *MEGF10* differential methylation in THC-exposed placentas.

We detected fewer differentially methylated CpGs with THC exposure in fetal tissues after multiple testing correction relative to placenta. This may be because placental DNA contains a higher frequency of partially methylated domains relative to blood and other tissues [57] and CpG sites with intermediate levels of methylation can be measured with greater reliability and precision than at extreme values (i.e., near *β* = 0 or fully unmethylated and near *β* = 1 or fully methylated) [58]. This may also be related to a lower relative THC exposure of fetal tissues compared with the placenta. However, the FDR DMCs identified in the fetal tissues warrant further investigation based on their biological relevance in those respective tissues. For example, in cerebellum we identified one hypomethylated CpG in the 3’UTR of neuron-specific gene family member 1 *(NSG1;* aka *NEEP21),* which is highly enriched in developing cerebellum [59]. Loss of this gene in mice is associated with increased anxiety-related behavior in certain tasks such as spending 50% less time in the open arms of the elevated plus maze [60]. In the right ventricle of the heart, we identified FDR significant hypomethylation of one CpG upstream of the heart and neural crest derivatives expressed 1 (*HAND1*) gene, which is essential to the formation of the right ventricle [61]. Additionally in the right ventricle, we observed hypermethylation of one CpG annotated to the neutral sphingomyelinase activation associated factor gene (*NSMAF)*, which may play a role in activation of neutral sphingomyelinase in response to cardiac ischemia or reperfusion injury [62].

We observed several placental DMRs annotated to a large block of protocadherin (*PCDH*) genes and individual CpGs with decreased methylation that were negatively correlated with increased expression of *PCDHB8* following THC exposure*.* Consistent with our observed effect of THC on placental methylation, a study of placental DNA methylation in autism cases versus control identified several differentially methylated CpGs in this region, the majority of which were hypomethylated [63]. The *PCDH* gene block is organized into 3 clusters containing *PCDHA*, *PCDHB*, and *PCDHG* genes, which are highly expressed in the developing nervous system where they play an important role in neuronal cellular diversity [64]. In mice, there is evidence that differential expression of *Pcdh* isoforms is regulated by differential methylation of their promoter regions [65], and in humans dysregulated *PCDH* methylation has been observed in several neurological and psychiatric disorders [66].

Our findings are also consistent with a recent study that used whole genome bisulfite sequencing to identify novel differentially methylated regions in placenta samples from a prospective ASD study. The study by Zhu et al. [67] included functional characterization of a novel transcript within a hypomethylated block at 22q13.33, renamed *NHIP* (neuronal hypoxia inducible, placenta associated). Within this region, we observed increased methylation and expression of *SHANK3*, a gene involved in glutamatergic synapse formation with known mutations, epigenetic dysregulation, and structural variations observed in patients with ASD and other neurological syndromes [68–71].

We recently reported that these THC-exposed pregnancies demonstrated findings suggestive of placental insufficiency including decreased amniotic fluid volume, placental perfusion, and fetal oxygen availability [72]. The placenta plays a vital role in overall fetal health, development, and growth through the supply of oxygen and nutrients, gas and waste exchange, and endocrine signaling [73, 74]. Impaired placental function can result in adverse offspring outcomes including growth restriction, stillbirth, and miscarriage [75]. The placenta also plays an important role in producing neurotransmitters that may directly affect fetal brain development, and there is growing evidence linking placental dysfunction with adverse neurobehavioral outcomes in offspring [76–78]. This intimate relationship between placental function and fetal brain development has led to the coinage of terms such as the “placenta-brain-axis” [79] and “neuroplacentology” [80].

To better understand the potential underlying mediators of our previous findings [72], in the current study we examined correlation of DNA methylation with THC-associated gene expression [72] and examined the potential functional relevance of these eQTM loci using network analysis. Out of 631 genes differentially expressed with THC exposure, 520 were associated with methylation at one or more CpG. Notably, placental eQTM genes were enriched for GO terms related to morphogenesis, response to wounding, and response to oxygen-containing compounds. Taken together, these results suggest that prenatal THC exposure dysregulates placental gene expression and function through effects on the epigenome, and these effects are also likely to impact brain development.

Several studies have also focused on using placental ‘omics to gain mechanistic insight into the relationship between the in utero environment and birth outcomes such as fetal growth, preterm delivery, and birthweight, and infant and childhood health outcomes such as neurocognition and behavior [81, 82]. As observed in our study from prenatal cannabis exposure, dysregulation of placental DNA methylation (DNAm) in response to the in utero environment is a common finding [81]. A prior literature review that included studies of placental DNAm and/or transcriptomics in response to the prenatal environment identified 28 articles meeting their search criteria. In 16 of these studies, there was evidence of mediation or biological plausibility linking these exposures to fetal and infant health outcomes through the placental epigenome [82]. More recently, the term “placenta epigenome–brain axis” was used to describe the relationship between disrupted placental function (linked to placental gene regulation) and neurocognitive function later in life [83]. Altogether, studies from this field of research suggest that neurobehavioral disorders such as ASD, likely originate with dysregulated placental function.

Although the current evidence is not sufficient to conclude that prenatal cannabis exposure is a cause or a risk factor for development of ASD in offspring, there are a few large cohort studies that examine long-term outcomes related to prenatal cannabis exposure longitudinally. A retrospective Canadian study linked pregnancy and birth data to provincial health administrative databases to ascertain child neurodevelopmental outcomes and found an association between maternal cannabis use in pregnancy and increased incidence of ASD in the offspring [30]. However, due to limitations in data availability, this study was not able to account for key risk factors that can increase the likelihood of developing ASD. Our study provides biological plausibility for the findings of increased likelihood of developing ASD reported by this large Canadian study.

Another recent study assessed the effects of maternal cannabis use on psychosocial and physiological measures in young children along with the potential relevance of the in utero environment reflected in the placental transcriptome [35]. This study identified a relationship between maternal cannabis use and transcriptome changes in the placenta as a potential mediator of risk for anxiety-related problems in early childhood. We have now identified DNA methylation as a potential mediator of gene expression changes following prenatal THC exposure related to placental function and offspring neurobehavioral development, which may be related to this study’s observations of increased offspring anxiety-related problems in early childhood with in utero cannabis exposure.

Strengths of our study are that it utilized a translational rhesus macaque model to overcome the limitations of existing studies such as the inability to measure cannabis exposure including timing, duration, frequency, dose, type of product, and administration route. Our study used weight-based THC dosing for rigor and reproducibility, and chose THC edibles as the mode of administration to recapitulate typical human THC use in pregnancy and to examine the direct effects of THC-only on the placental and fetal epigenome without other confounders. Additionally, our study also overcomes the paucity of human epigenetic studies on prenatal cannabis exposure due to lack of feasibility in obtaining fetal tissue and ethical challenges related to research on maternal use of federally illegal drugs, such as cannabis. We were able to extend our investigation beyond the placenta to include 4 additional fetal tissues, not accessible in human pregnancies, for comparison of epigenetic signatures in response to prenatal THC exposure. All placental and fetal tissue were collected at time of cesarean section delivery from pregnancies with similar environmental exposures, including diet. This controlled tissue collection minimizes potential confounders, such as inflammation. We utilized the Illumina MethlyationEPIC platform in order to allow for direct comparison of our results with those from human studies. Future studies should consider whole genome or reduced representation bisulfite sequencing in order to identify potentially novel loci not covered by MM valid EPIC probes. Additionally, this study was limited by a smaller cohort size, which did not provide the power to examine fetal sex as a biological variable.

## Conclusions

Ultimately, the long-term consequences of the altered placental and fetal epigenome that we report are unknown, but may impact offspring development and health. The findings of this study add to the limited safety data on prenatal cannabis exposure and provide potential underlying mechanisms, such as placental dysfunction and altered placental DNA methylation, for the observations of altered offspring neurobehavior associated with maternal cannabis use in pregnancy reported in the literature. Taken together, our findings lay the groundwork for ongoing postnatal studies focused on determining the impact of chronic prenatal and postnatal THC exposure on infant neurodevelopment, sensorimotor development and socioemotional behavior in the rhesus macaque. As the prevalence of prenatal cannabis use is rising along with significant increases in potency, there is an urgent need for evidence-driven recommendations on the safety of use both prenatally and postnatally. The data from this study bridge this current gap in knowledge to help guide patient counseling and public health polices focused on cannabis use.

## Materials and methods

### Experimental design

This study used indoor-housed sexually mature, female rhesus macaques (*n* = 10) maintained on a standard chow diet (TestDiet, St. Louis, Missouri) with an additional cookie with either THC (*n* = 5) or placebo (*n* = 5) daily. Cookies containing THC (THC edible) were made using research-grade THC obtained directly from the National Institute on Drug Abuse (NIDA) Drug Supply Program [40, 41]. Placebo cookies were identical to those containing THC except without the THC-vehicle. Tap water was available ad libitum. Edibles were administered prior to the animal’s daily chow to ensure consumption on an empty stomach and to confirm complete ingestion. Animals were slowly titrated up to 2.5 mg/7 kg/day of THC using published weight-based medical cannabis acclimation recommendations [72] approximately 4 months prior to undergoing time-mated breeding as previously published [40]. We previously established [40, 41] that NHP peak THC levels are within the expected reported contemporary dosing range (e.g., 5–8 ng/mL) in humans 3 h following a similar oral THC dose [84, 85]. Each THC-exposed pregnant animal (*n* = 5) continued to consume a daily THC edible of 2.5 mg/7 kg/day throughout pregnancy. All animals (*n* = 10) underwent immediate cesarean section delivery with placental collection and fetal necropsy on gestational day 155 (term is ~ 168 days) to minimize confounding variables secondary to spontaneous labor. Collected placental tissue was processed in RNAlater for RNA-sequencing. Placental tissue, in addition to fetal lung, right ventricle of the heart, prefrontal cortex, and cerebellum tissues were also collected and flash-frozen in liquid nitrogen with storage at − 80 °C for DNA studies. All protocols were approved by the Oregon National Primate Research Center (ONPRC) Institutional Animal Care and Use Committee and conformed to all guidelines for humane animal care (IP0001389).

### DNA extraction and DNA methylation (DNAm) array acquisition and preprocessing

Placental and fetal tissue DNA was extracted using the AllPrep miRNA kit (Qiagen) and the QIAcube for automated nucleic acid extraction (Qiagen, USA). Epigenome-scale DNAm was measured with the Infinium MethylationEPIC BeadChip (Illumina, San Diego, California) at the Fred Hutchinson Cancer Genomics Resource (Seattle, WA). Processed BeadChips were scanned using the Illumina iScan + with ICS v3.3.28, and intensity data were extracted with Illumina GenomeStudio software (GenomeStudio v2011.1 with Methylation Analysis Module v1.9.0). All analysis was performed using R statistical software (version 4.1.1). Data normalization and QC were performed using the *minfi* [86] and *ChAMP* [87] packages. We examined density plots of beta values at each preprocessing and filtering step. Stratified quantile normalization preprocessing was performed using *minfi* to adjust for probe-type bias in beta distributions. Probes previously determined to be functional and valid in *Macaca mulatta* (referred to as MM_valid) [48] were retained prior to removing probes with failed detection *p* value > 0.01 in ≥ 25% of samples. For each tissue, we visually inspected the median intensity of measurements on the X and Y chromosome and confirmed that predicted sex agreed with biological sex. In addition, for each tissue, we performed hierarchical clustering and multidimensional scaling (MDS) plots to check that technical duplicates clustered together. Duplicate samples were removed prior to downstream analysis. Lastly, we removed probes mapping to non-autosomal chromosomes and probes with a colony SNP allele frequency > 0.1 at the CpG of interest [88]. We also identified common SNPs overlapping probe regions not at the CpG site, which could potentially influence probe binding or estimated DNA methylation.

### Analysis of differentially methylated CpGs (DMCs) and differentially methylated regions (DMRs) between treatment groups

We performed differential methylation analysis using *limma* [89] with methylation for each CpG site as the response variable on the M-scale (logit2 beta). Given that our samples came from the same set of animals, we first performed within subject correlation across tissues using the dupCor function. We then fit CpG-specific models that included a factorial design for group and subject, a blocking term for subject, and the inter-animal correlation value. We used quantile–quantile plots of *p* values to visualize genomic inflation and computed estimated coefficients and standard errors separately for each tissue. We also calculated the mean beta values per CpG and the delta beta for each tissue and treatment group (mean THC—mean CON), for more intuitive biological interpretation. We identified DMRs using the DMRcate package, and the same statistical design used for DMC analysis and tissue-specific contrasts. DMRcate computes a kernel estimate against a null distribution. We used the default lambda setting of 1000 bp to bookend DMRs (i.e., CpGs > 1000 bp apart are split into separate DMRs) and FDR = 0.05 cutoff threshold to determine DMR significance [90].

### Enrichment analysis of biological pathways and gene ontology (GO) terms

We performed enrichment analysis of DMCs with nominal *p* value < 0.05 for each tissue separately using the missMethyl package [91], which accounts for bias due to either (1) a greater number of probes per gene covered in the array dataset, or (2) CpGs which are annotated to multiple genes. As background for enrichment, we used the genes annotated to our filtered MM_valid probe set. For each tissue and set of nominally significant DMCs, we tested for enrichment of GO terms and KEGG pathways, separately. In the placenta only, we performed GO enrichment analysis using the ClusterProfiler package to test for and visualize enrichment of FDR significant DMCs [92].

### Expression quantitative trait methylation (eQTM) analysis

We performed expression quantitative trait methylation (eQTM) analysis using the *MEAL* package for correlation of expression and methylation [93]. Genome-wide RNA-sequencing and EPIC DNAm was available for 10 placental and 10 lung samples. We used the *biomart* package to convert *mmulatta* ensembl IDs to *hsapiens (version 75)* [94]. We used the default flanking parameter to identify CpGs located within 250 kb of the transcription start site (TSS) of differentially expressed mRNA transcripts.

### Enrichment analysis of candidate autism disorder genes

We used the publicly available SFARI database [52] to generate a list of genes previously implicated in autism risk. We compared the overlap of candidate autism genes with genes annotated to CpGs differentially methylated with THC (*p* < 0.05) for each tissue using Venn diagrams and the hypergeometric test for significant overlap. Enrichment *p* values < 0.05 were considered significant. As a negative control, and to ensure that the MM_valid probes were not biased for candidate ASD genes, we also performed enrichment testing at multiple *p* value thresholds and compared our results to enrichment *p* values for randomly selected gene sets of the same size [54]. We next compared our list of genes differentially methylated with THC in rhesus placenta to a published list of DMR genes in human placentas from pregnancies where the newborn was later diagnosed with ASD using the hypergeometric test for significant overlap [53].

## Supplementary Information

**Additional file 1**. **Figure S1**. Density and MDS plot of quantile normalized beta values by tissue type; **Figure S2**. Tissue specific QQ-plots and inflation measures; **Figure S3**. MDS plot of most variable probes in placenta colored by treatment group; **Figure S4**. Boxplots of FDR significant DMCs in fetal tissues; **Figure S5**. Visual summary of placental methylation and expression within protocadherin gene cluster.Gviz plot showing genomic ranges, significant eQTM correlation coefficients, average difference in methylation in THC versus CON animals, and average methylation per CpG. On the “Difference THC” track, DMCs are shown in grey and DMRs are shown in red or blue indicating hyper or hypomethylation, respectively.Boxplot of *PCDHB8* expression.Top CpG associated with *PCDHB8* expression; **Figure S6**. Permutation testing with 10 random gene sets the same size as the SFARI candidate ASD gene list were not enriched among DMC genes; **Figure S7**. STRING protein–protein interaction plot of placental genes with significant differential expression between THC and CON animals and correlation with methylation at one or more CpG. We retained protein with high confidence interaction and color performed k-means clustering into 3 clusters, indicated by color of each bubble; **Figure S8**. Visual summary of placental methylation and expression within *SHANK3* gene region.Gviz plot showing genomic ranges, significant eQTM correlation coefficients, average difference in methylation in THC versus CON animals, and average methylation per CpG. On the “Difference THC” track, DMCs are shown in red or blue indicating hyper or hypomethylation, respectively.Boxplot of *SHANK3* expression.Top CpG associated with *SHANK3* expression;

**Additional file 2**. **Table S1.** DMCs associated with prenatal THC exposure at FDR significance in one or more tissues; **Table S2.** DMRs associated with prenatal THC exposure at FDR significance in placenta; **Table S3**. MissMethyl FDR significant GO and KEGG Terms; **Table S4**. Summary of SFARI genes which overlap with THC DMC genes; **Table S5**. Significant eQTM for placental differentially expressed genes sorted by genomic coordinates using EPIC annotation; **Table S6.** Placental eQTM gene FDR significant GO Terms from STRINGdb

## Data Availability

The raw and processed DNA methylation and RNA-seq datasets used and/or analyzed during the current study are publicly accessible through NCBI Gene Expression Omnibus (GEO) via accession series  GSE223825.

## Abbreviations

THC: Delta-9-tetrahydrocannabinol
ASD: Autism spectrum disorder
ADHD: Attention deficit hyperactivity disorder
SFARI: Simons Foundation Autism Research Initiative
NHP: Non-human primate
FDR: False discovery rate
DMCs: Differentially methylated CpGs
PFC: Prefrontal cortex
SNP: Single nucleotide polymorphism
GO: Gene ontology
KEGG: Kyoto Encyclopedia of Genes and Genomes
eQTM: Expression quantitative trait methylation
DE: Differentially expressed
DMRs: Differentially methylated regions
ONPRC: Oregon National Primate Research Center
MDS: Multidimensional scaling
TSS: Transcription start site
