# Supplementary material for: Prenatal delta-9-tetrahydrocannabinol exposure is associated with changes in rhesus macaque DNA methylation enriched for autism genes
Source: Clin Epigenetics. 2023 Jul 6;15:104. doi: 10.1186/s13148-023-01519-4 (PMC10324248; doi:10.1186/s13148-023-01519-4)
Supplement: Supplementary file 1 — Additional file 1. Figure S1. Density and MDS plot of quantile normalized beta values by tissue type; Figure S2. Tissue specific QQ-plots and inflation measures; Figure S3. MDS plot of most variable probes in placenta colored by treatment group; Figure S4. Boxplots of FDR significant DMCs in fetal tissues; Figure S5. Visual summary of placental methylation and expression within protocadherin gene cluster.Gviz plot showing genomic ranges, significant eQTM correlation coefficients, average difference in methylation in THC versus CON animals, and average methylation per CpG. On the “Difference THC” track, DMCs are shown in grey and DMRs are shown in red or blue indicating hyper or hypomethylation, respectively.Boxplot of PCDHB8 expression.Top CpG associated with PCDHB8 expression; Figure S6. Permutation testing with 10 random gene sets the same size as the SFARI candidate ASD gene list were not enriched among DMC genes; Figure S7. STRING protein–protein interaction plot of placental genes with significant differential expression between THC and CON animals and correlation with methylation at one or more CpG. We retained protein with high confidence interaction and color performed k-means clustering into 3 clusters, indicated by color of each bubble; Figure S8. Visual summary of placental methylation and expression within SHANK3 gene region.Gviz plot showing genomic ranges, significant eQTM correlation coefficients, average difference in methylation in THC versus CON animals, and average methylation per CpG. On the “Difference THC” track, DMCs are shown in red or blue indicating hyper or hypomethylation, respectively.Boxplot of SHANK3 expression.Top CpG associated with SHANK3 expression; [file 13148_2023_1519_MOESM1_ESM.pptx]

## Slide 1
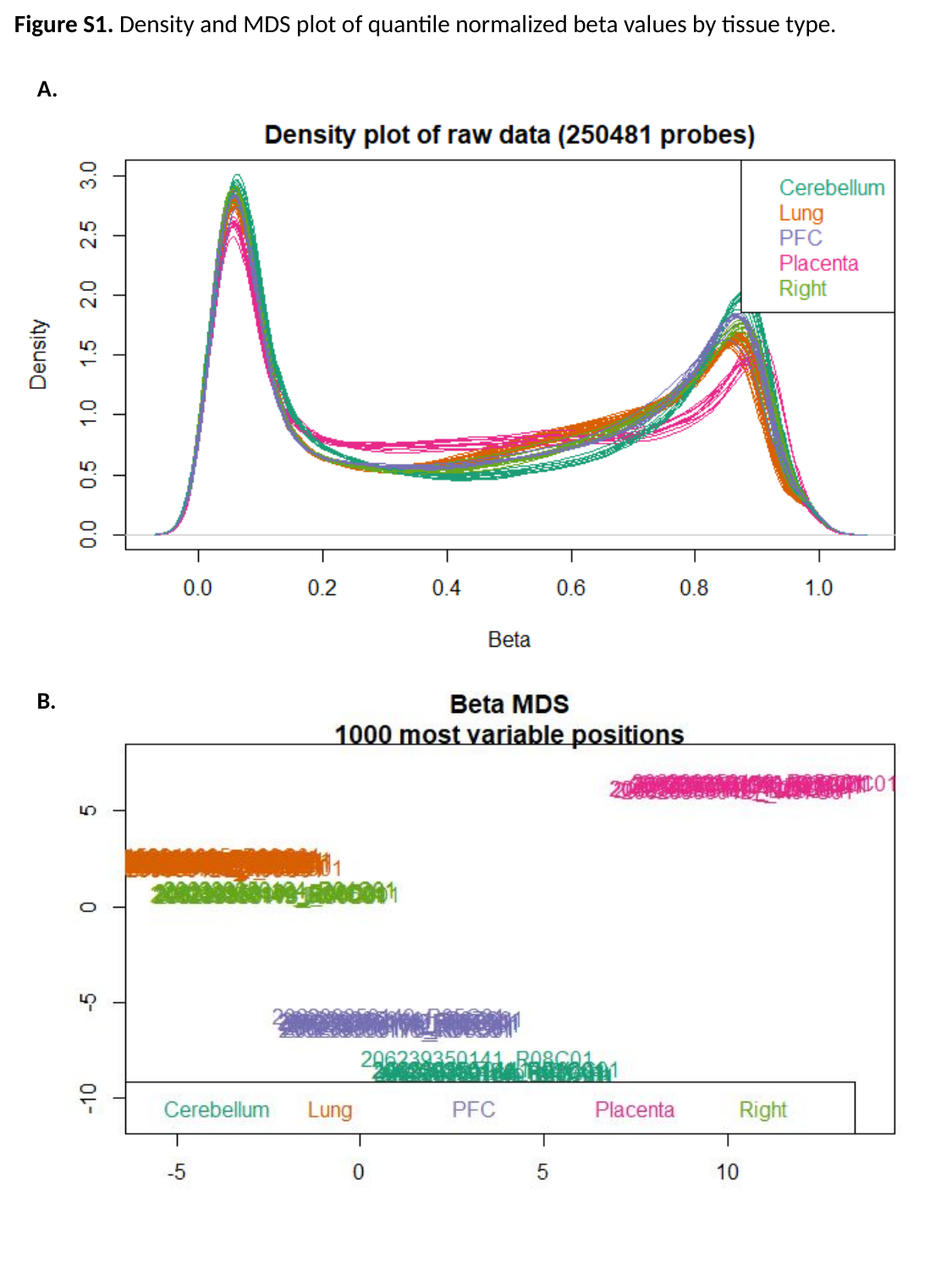

Figure S1. Density and MDS plot of quantile normalized beta values by tissue type.
A.
B.
B.

## Slide 2
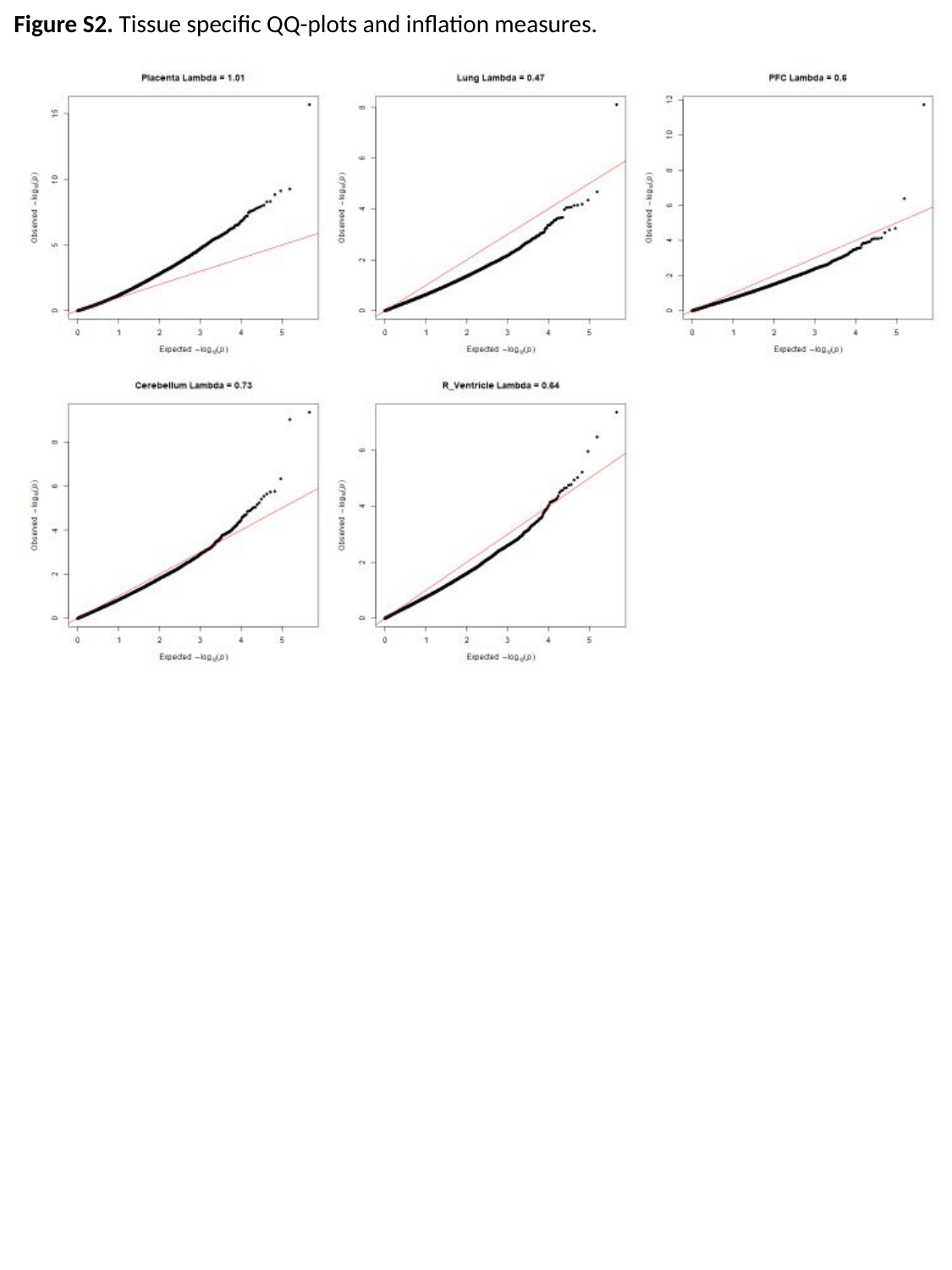

Figure S2. Tissue specific QQ-plots and inflation measures.

## Slide 3
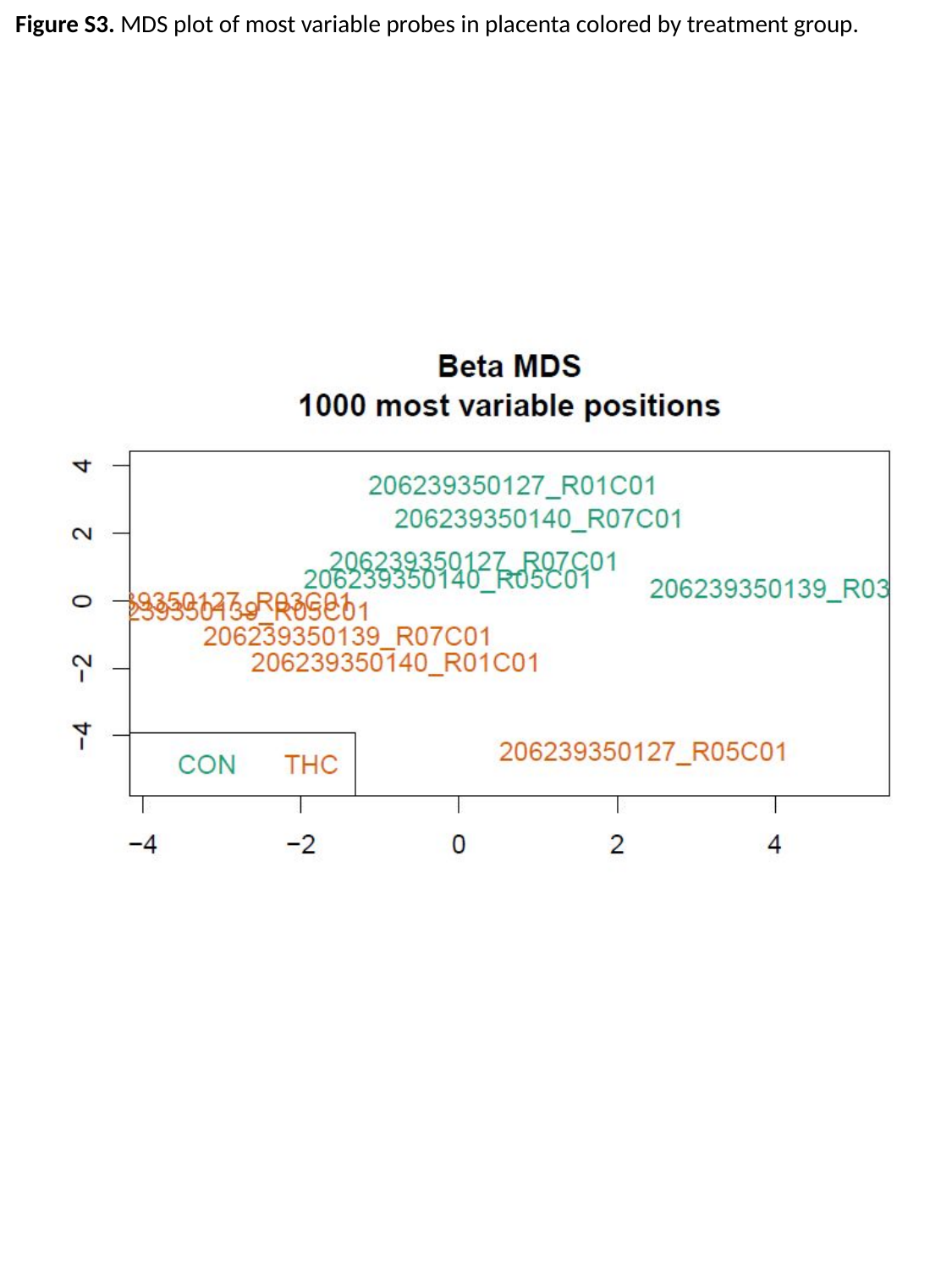

Figure S3. MDS plot of most variable probes in placenta colored by treatment group.

## Slide 4
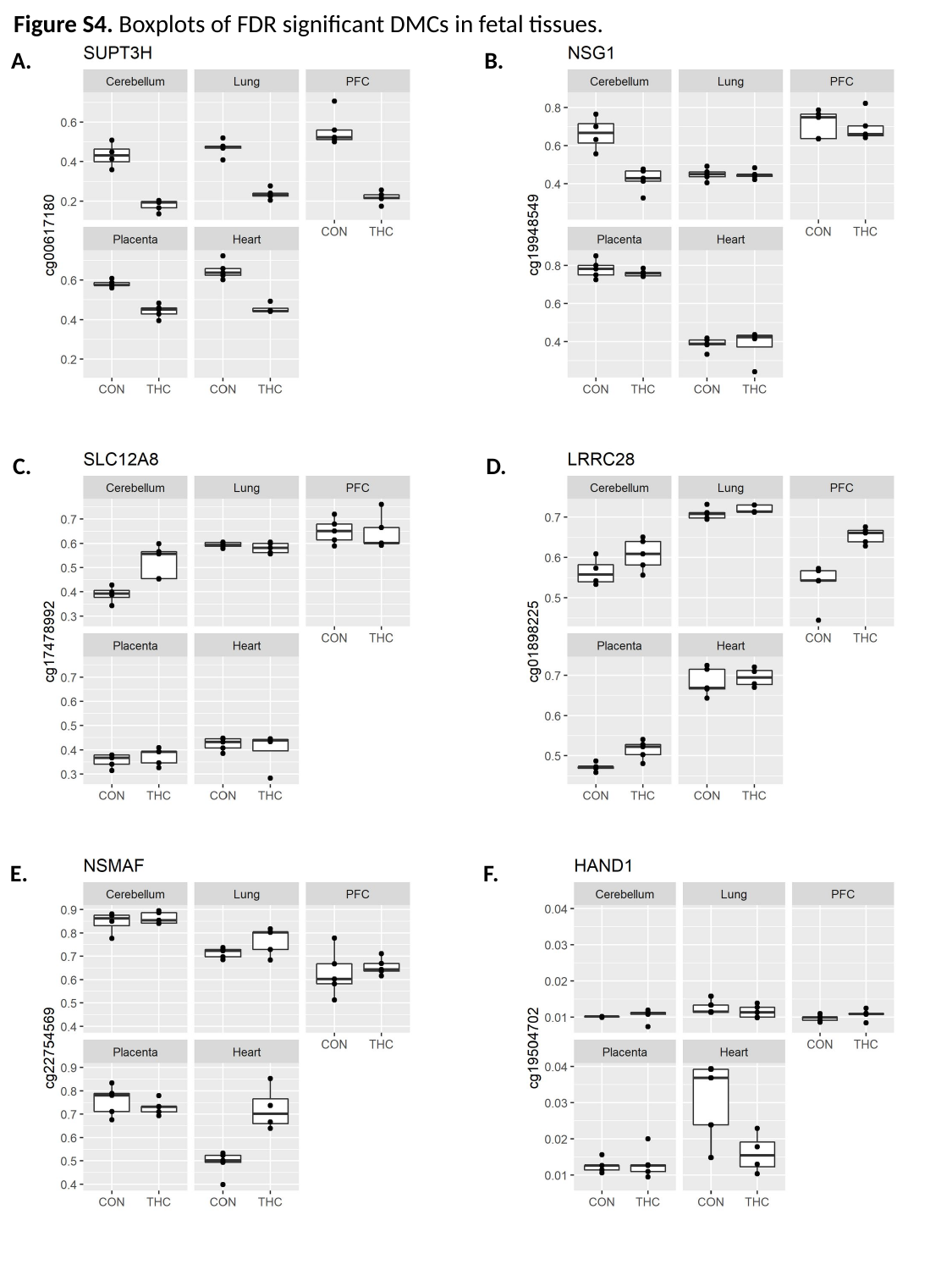

Figure S4. Boxplots of FDR significant DMCs in fetal tissues.
A.
B.
C.
D.
E.
F.

## Slide 5
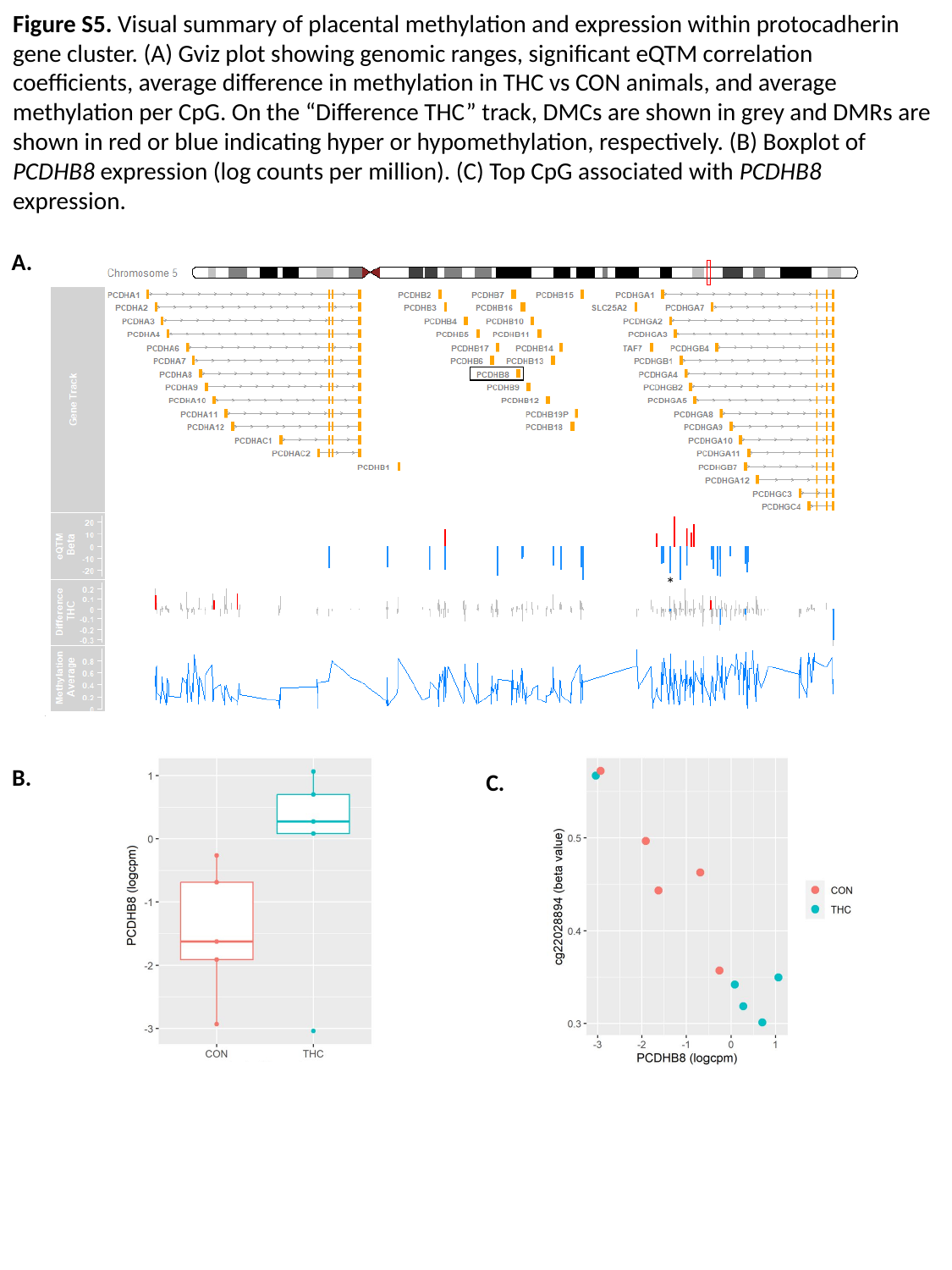

Figure S5. Visual summary of placental methylation and expression within protocadherin gene cluster. (A) Gviz plot showing genomic ranges, significant eQTM correlation coefficients, average difference in methylation in THC vs CON animals, and average methylation per CpG. On the “Difference THC” track, DMCs are shown in grey and DMRs are shown in red or blue indicating hyper or hypomethylation, respectively. (B) Boxplot of PCDHB8 expression (log counts per million). (C) Top CpG associated with PCDHB8 expression.
A.
*
B.
C.

## Slide 6
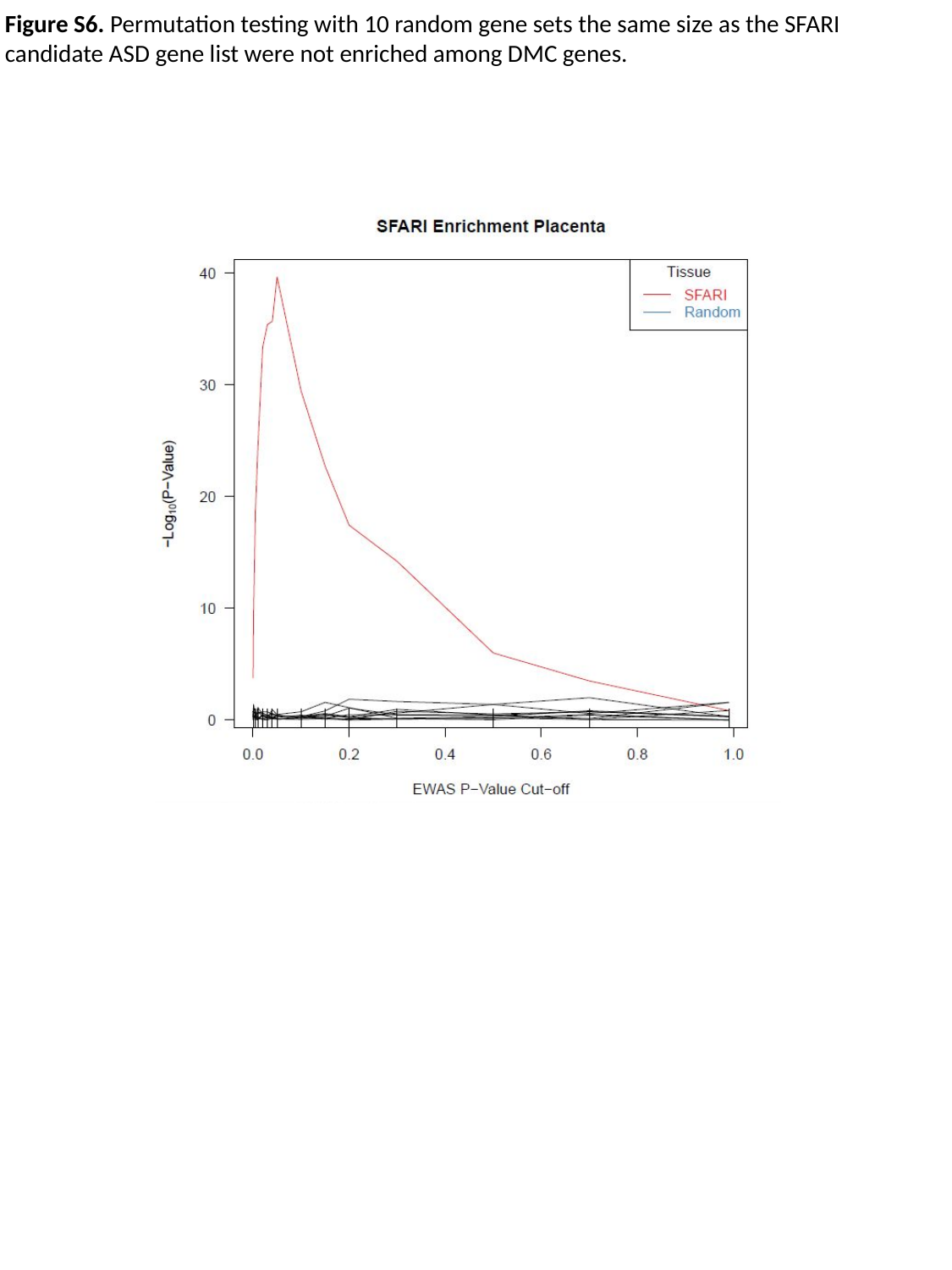

Figure S6. Permutation testing with 10 random gene sets the same size as the SFARI candidate ASD gene list were not enriched among DMC genes.

## Slide 7
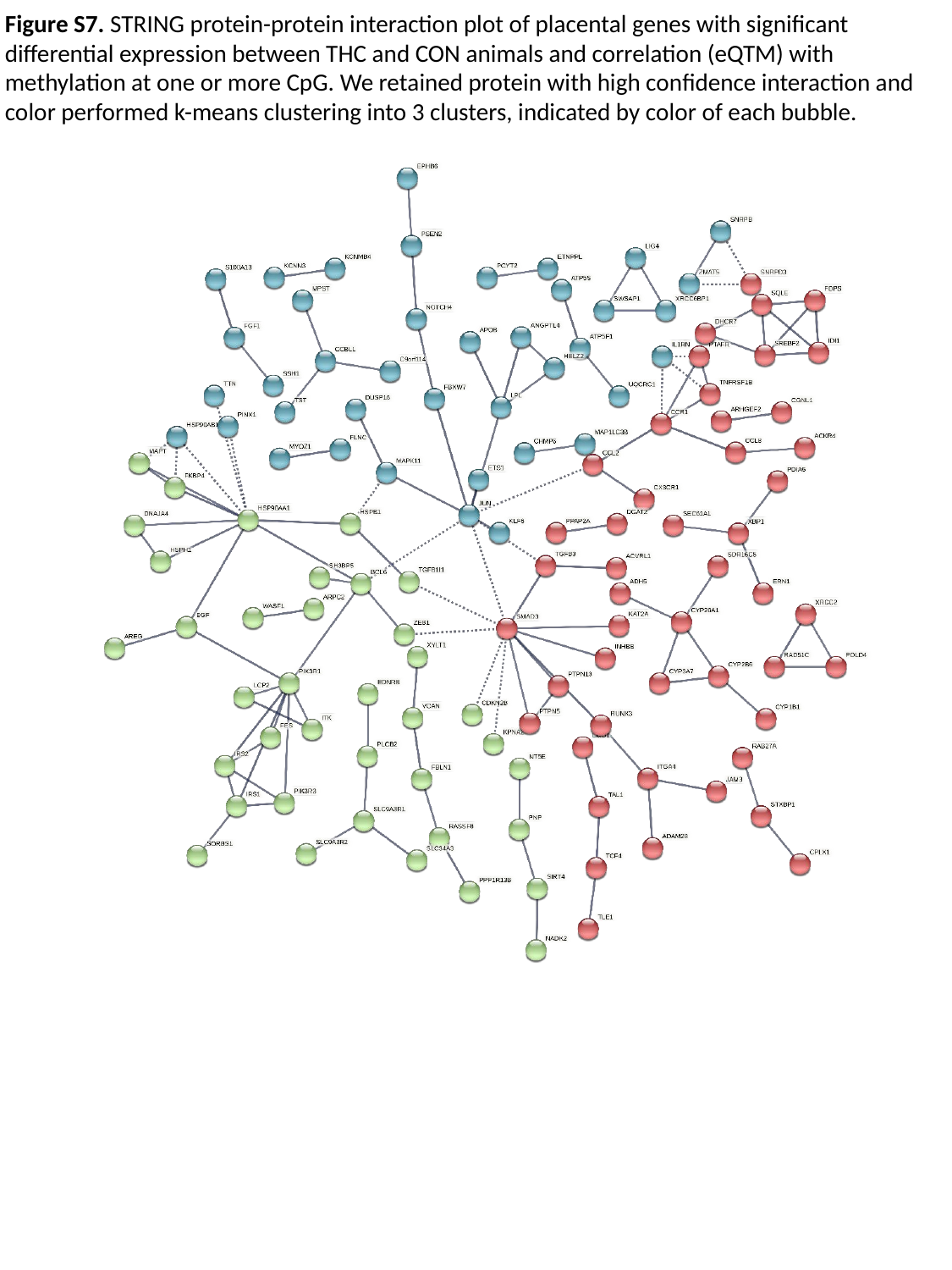

Figure S7. STRING protein-protein interaction plot of placental genes with significant differential expression between THC and CON animals and correlation (eQTM) with methylation at one or more CpG. We retained protein with high confidence interaction and color performed k-means clustering into 3 clusters, indicated by color of each bubble.

## Slide 8
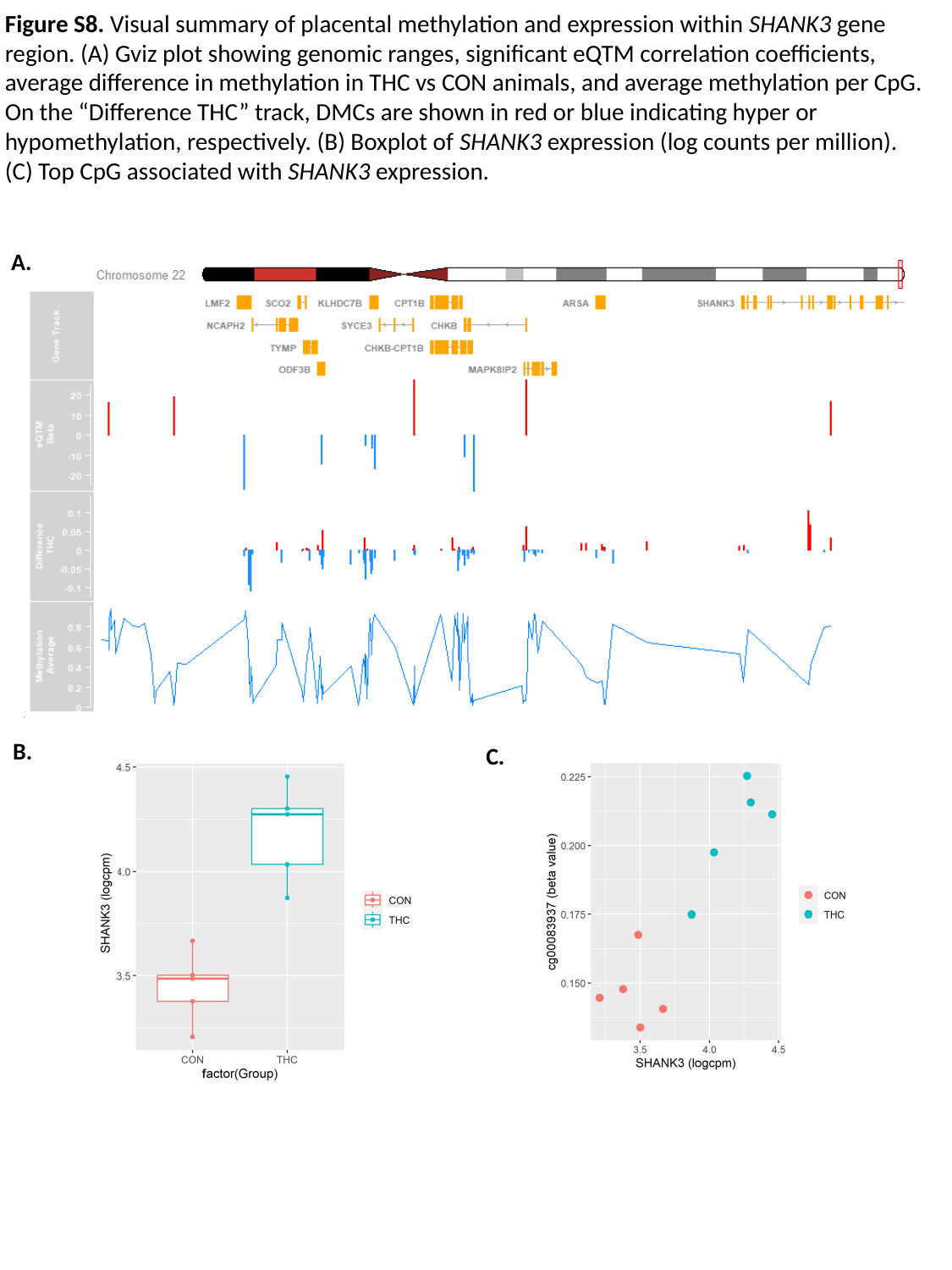

Figure S8. Visual summary of placental methylation and expression within SHANK3 gene region. (A) Gviz plot showing genomic ranges, significant eQTM correlation coefficients, average difference in methylation in THC vs CON animals, and average methylation per CpG. On the “Difference THC” track, DMCs are shown in red or blue indicating hyper or hypomethylation, respectively. (B) Boxplot of SHANK3 expression (log counts per million). (C) Top CpG associated with SHANK3 expression.
A.
B.
C.
